# Impact Printing

**DOI:** 10.1089/3dp.2021.0068

**Published:** 2022-06-09

**Authors:** Coralie Ming, Ammar Mirjan, Jesús Medina Ibáñez, Fabio Gramazio, Matthias Kohler

**Affiliations:** Chair of Architecture and Digital Fabrication, ETH Zurich, Zurich, Switzerland.

**Keywords:** Impact Printing, material shooting, soft particles, graded materials

## Abstract

This article introduces the concept of Impact Printing, a new additive manufacturing (AM) method that aggregates malleable discrete elements (or soft particles) by a robotic shooting process. The bonding between the soft particles stems from the transformation of kinetic energy, gained during the acceleration phase, into plastic deformation upon impact. Hence, no additional binding material is needed between the soft particles; the cohesion and self-interlocking capacities of the material itself acts as the primary binding agent. Shooting, and consequent impacting, forces can be modulated and result in distinct compaction ratios. By linearly shooting material, we decouple the deposition apparatus from the produced parts and provide flexibility to the deposition process to potentially build in any directions or onto uncontrolled surfaces. Impact Printing produces parts with formal characteristics standing between brick laying—assembly of discrete building blocks—and 3D Printing—computer-controlled depositioning or solidifying of material. It brings forward a novel digital fabrication method and an alternative to the conventional continuous AM process. This article validates the Impact Printing approach with a series of prototypical experiments, conducted with a robotic fabrication setup consisting of a six-axis robotic arm mounted with a material shooting apparatus, that forms, orients, and projects the soft particles. We will explain and demonstrate its principles and define the fabrication parameters, such as shooting force, shooting distance, and the resulting aggregations' characteristics.


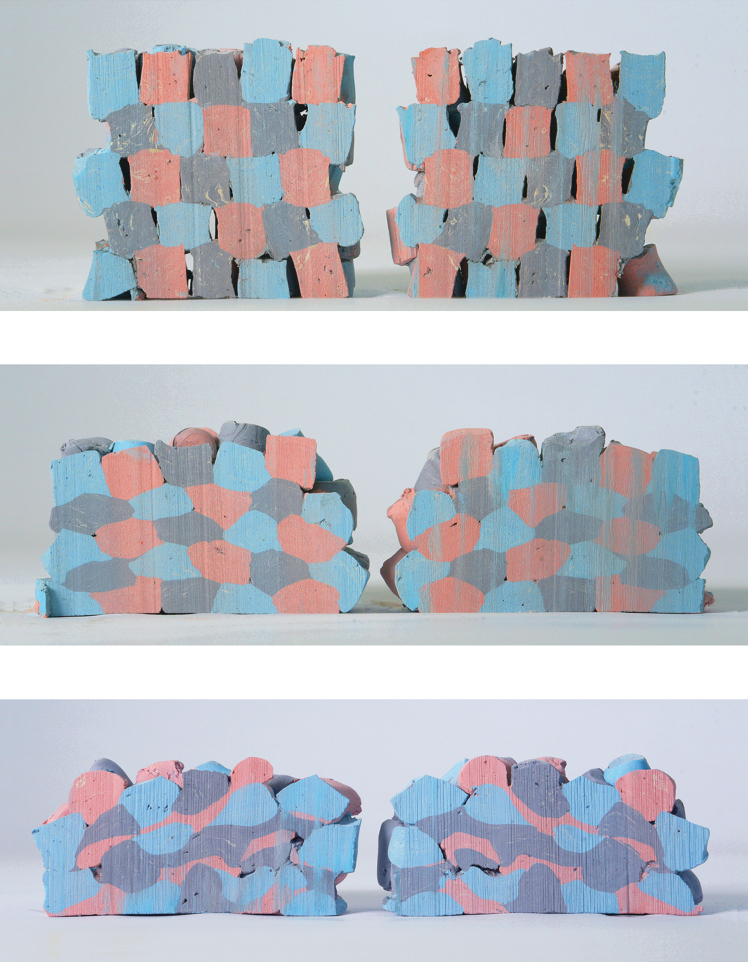


## Introduction

### 3D printing methods

Additive manufacturing (AM) technologies enable the construction of standard or nonstandard structures^1–4^ through distinct material deposition methods. The most common are *material extrusion*, where material is selectively dispensed through a nozzle, *binder jetting*, and *powder bed fusion*, where a liquid bonding agent or thermal energy selectively joins powder materials, *directed energy deposition*, where focused thermal energy is used to fuse materials by melting during deposition,^2^ and *material aggregation*, where solid material is selectively aggregated.

These AM methods range from fully continuous (material extrusion)^3–6^ to fully discrete^4^ material deposition processes. They operate at a wide range of scales; from a particle level (binder jetting, powder bed fusion) enabling the production of high-resolution parts,^5,6^ to the aggregation of larger building elements resulting in the coarser construction of full-scale structures (material aggregation).^7,8^

Typically, AM machines are based on linear or rotary motions along axes to physically transport and deposit material in a layering process.^9^ They are position control systems, leading to high-precision manufacturing, but also limiting the scale of the produced parts to the physical building work range of the robot. Such constraints could potentially be overcome by an alternative mode of material transport: flying, enabled by shooting mechanisms.

### Remote aggregations

If mechanized shooting as a means to transport and aggregate material has not yet been adopted by production systems, nor the building industry, manual throwing has a long-lasting history in building processes. Cob walls for instance, are aggregated by manually throwing from human height, small portions of an earthen compound in a layering sequence.^10^ When hydrated, the material is in a malleable state enabling the impacting aggregates to deform and bind to one another, gradually building-up architectural-scale structures. Thus, cob sets the premises of a discrete deposition method to aggregate malleable material through a throwing process.

**FIG. 1.**
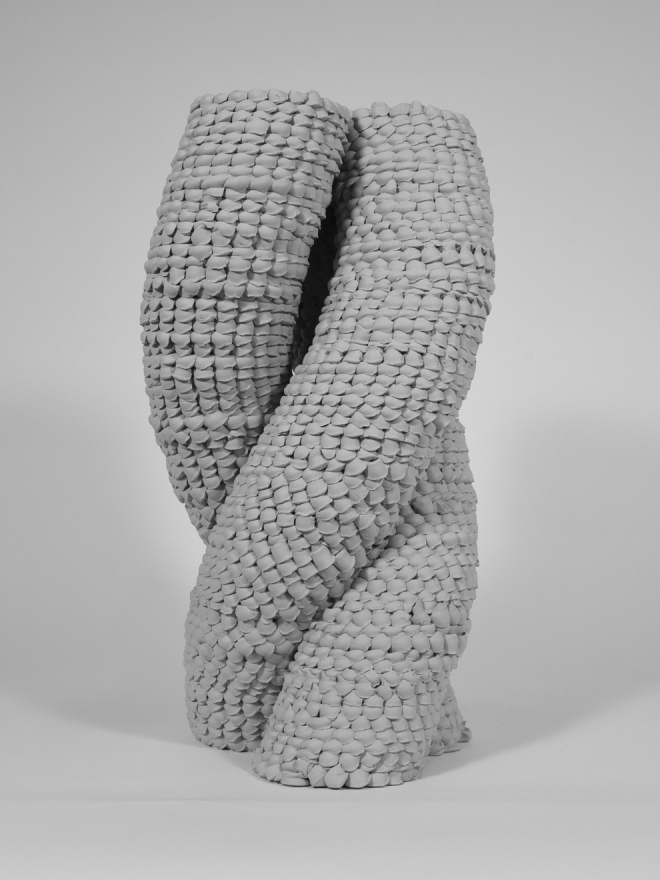
Photo of a geometrically complex prototype produced by Impact Printing. Design and fabrication by the students of the MAS DFAB, ETH Zurich: Hakim Hasan and Rodrigo Diaz Escalante. Image © Gramazio Kohler Research, ETH Zurich. Reprinted with permission.

Recently, in a contemporary reinterpretation of cob, the architectural installation *Remote Material Deposition (RMD)*,^11^ by Gramazio Kohler Research, ETH Zurich, took the first step toward the implementation of mechanized throwing as an AM method. A small robotic system threw malleable clay-based* cylinders—or soft particles—over large distances (up to 14.00 m) and deposited material following a ballistic curve. RMD proved that the mechanized throwing is a valid material deposition method applied to construction. It showcased advantages such as high reach and flexibility, but also came with an intrinsic lack of control over the thrown soft particles.^12^ Additionally, the geometrical freedom of RMD was limited due to the soft particles' ballistic curve consistently impacting the building space vertically. With Impact Printing, we propose a higher level of position control (Fig. 1) by increasing the speed of the projectile until reaching a linear motion, enabling the deposition process to follow not only a horizontal layering, but also potentially all directions.

### Introduction to Impact Printing

Impact Printing takes a first step toward the implementation of mechanized linear shooting into an AM process. It differs from existing technologies by, *first*, being a contactless AM process: the deposition apparatus is separated from the built structure and the length of the shooting range increases the reach of the robot. As in RMD, structures being bigger than the robotic setup can be built, eliminating the need for an oversized machinery. *Second*, it can be a speedy AM process by replacing part of the lengthy kinematic motions required to physically transport material by a fast material shooting process. Additionally, it can aggregate discrete elements, potentially as large as standard construction bricks, enabling the efficient building of architectural-scale structures. *Third*, the impact results in a local ramming process, where an interlocking bond emerges between the soft particles. This impacting force can be modulated to enable graded design and the integration of building add-ons or structural requirements. Therefore, by presenting the Impact Printing concept, this article brings forward a novel digital fabrication method.

## Materials and Methods

### Overarching principles

Impact Printing is an AM method that uses kinetic energy (*E*_K_), or the energy possessed by a projectile due to its motion, to transport and plastically deform material upon impact. In this study, the soft particles gain their *E*_K_ during the acceleration phase where they transition from a state of rest to a flying motion. During their flying phase, the soft particles maintain most of their *E*_K_, until the impact, where nearly the same amount of energy is transformed to decelerate until reaching again a state of rest. There, *E*_K_ is converted into plastic deformation and surface cohesion resulting in the unification of both impacted and impacting soft particles. *E*_K_ formula (excluding air friction and gravity) showcases that the amount of energy results from the square of the projectile velocity, meaning that the amount of energy transferred upon impact exponentially increases in relation to a linear increase in speed: projectile mass is *m* (kilogram), velocity is *v* (meter/second): *E*_K_ = 1/2 *mv*^2^.

### Material characterization

Impact Printing requires specific material properties for the successful aggregation and bonding of its soft particles. Unlike other discrete material deposition processes (i.e., masonry structures) that are typically bound with a secondary material (mortar), Impact Printing uses the self-interlocking capacities and cohesion of the material itself as the primary binding agent. Thus, the material is required: (1) to have a plastic or viscoplastic behavior, meaning to be an inelastic material maintaining a deformation. (2) To enable surface cohesion between the aggregates to ensure a good bond. (3) To exhibit a malleability being equally firm—to maintain shape during acceleration—and soft—to allow for plastic deformation upon impact. (4) To have the capacity to cure and transition from a malleable to a hard state, with constant volume to avoid cracks and failures.

Potential suitable candidates include (but are not limited to): paste-like materials such as cement, concrete, and some polymers; cellulose slurries; or earthen compounds. We selected clay for its recyclable, sustainable and scalable qualities. We use a Swiss local product: B128 produced by Bodmer Ton AG (Einsiedeln, Switzerland).^13^ Typically used for pottery, the product mainly contains a mix of Silica (SiO_2_) and Alumina (Al_2_O_3_),^†^ water, and an additional 25% of fine grog (0–0.2 mm) to minimize shrinkage during the drying process.

### Robotic setup and Impact Printing apparatus

To produce the prototypes, we develop a robotic fabrication setup to ensure a precise and repeatable process. The setup consists of a table-mounted Universal Robot UR5 six-axis robotic arm customized with a digitally controlled material shooting apparatus mounted to the end of the robotic arm. Computer control enables an iterative robotic building sequence of (1) loading of projectile, (2) orientation of the robot to its shooting position, and (3) releasing of compressed air into the barrel to shoot the projectile toward the target position.

The developed soft particle shooting apparatus consists of a compressed air gun. It is essentially a straight tube where the compressed air inlet, controlled by a pressure regulator and a digitally activated solenoid multivalve block, is connected to its back end. When activated, the compressed air rapidly expands in the acceleration chamber and shoots the soft particle out. To enable a good seal and provide maximum efficiency during the acceleration phase, the soft particles have no encapsulating layer and are in direct contact with the tube's inner surface. Thus, the tube's material must be carefully selected to minimize the friction with the projectile during acceleration: acrylic, PVC, and aluminum obtained the best sliding efficiencies to propel the selected clay soft particles.

**FIG. 2.**
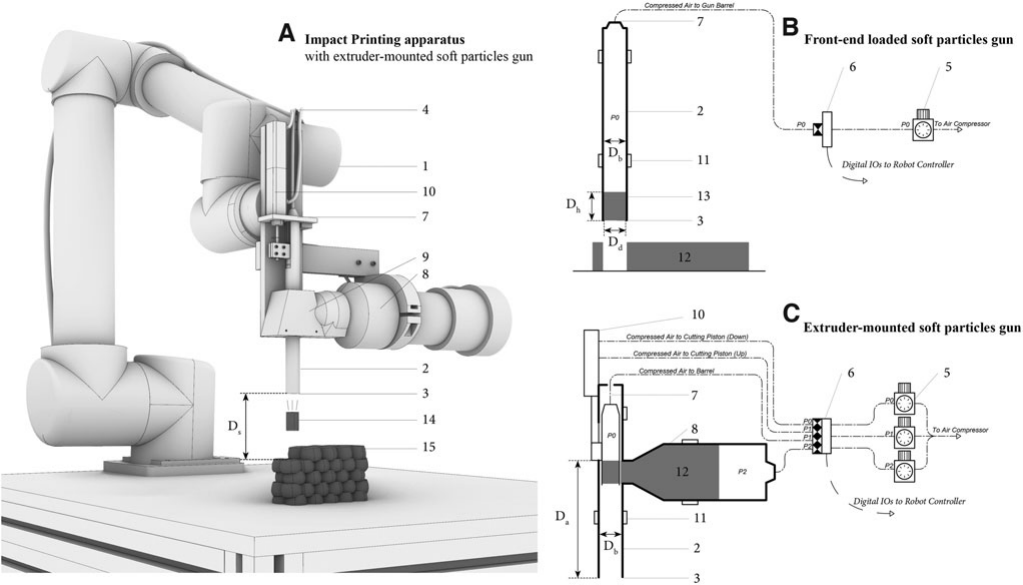
**(A)** Render view of an Impact Printing apparatus during fabrication, schematic section through **(B)** front-end-loaded soft particles gun and **(C)** extruder-mounted soft particles gun. The illustrations present the following elements: (1) UR5 six-axis robotic arm, (2) barrel, (3) barrel front-end, (4) compressed air inlet, (5) air pressure regulator, (6) digitally controlled valve, (7) compressed air inlet to barrel, (8) material extruder, (9) connecting chamber between extruder and barrel, (10) piston for in-barrel material cutter, (11) connector to robotic arm, (12) malleable material, (13) in-barrel projectile, (14) in-flight projectile, (15) impact printed prototype (*D*_s_) shooting distance (*D*_h_) soft particle height before impact, (*D*_d_) soft particle diameter before impact, (*D*_b_) barrel diameter, (*D*_a_) distance of in-barrel acceleration. Image © Gramazio Kohler Research, ETH Zurich. Reprinted with permission.

**FIG. 3.**
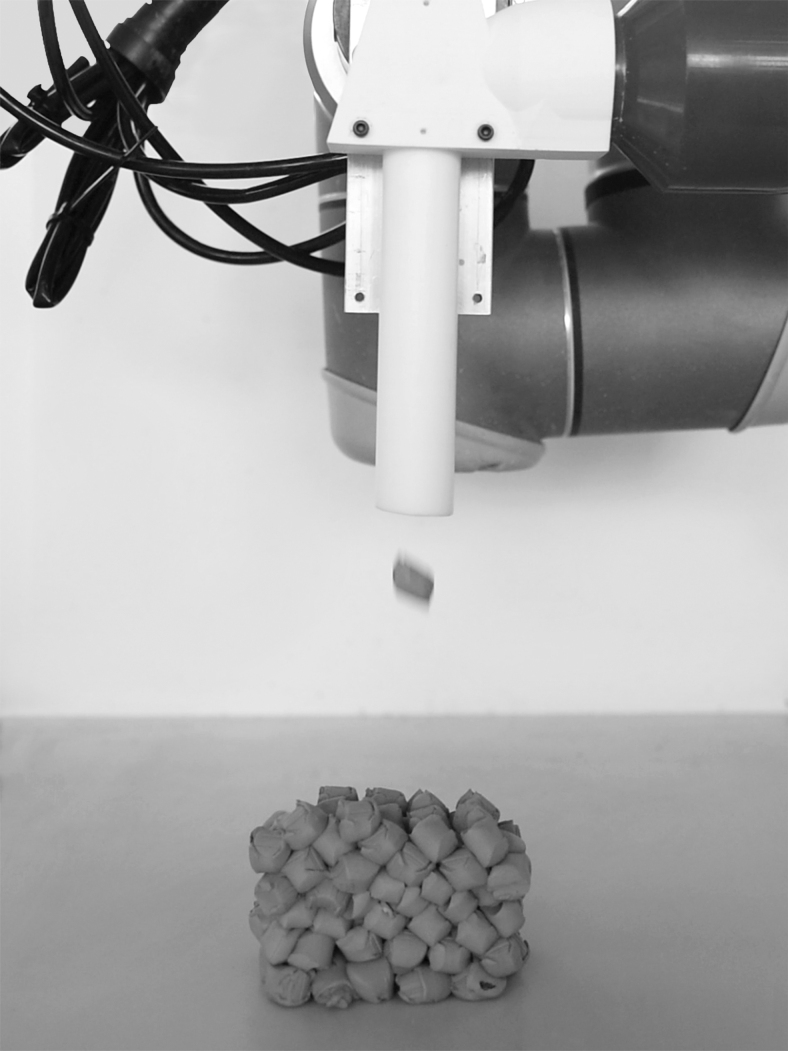
Photo of an Impact Printing process during fabrication. Image © Gramazio Kohler Research, ETH Zurich. Reprinted with permission.

We developed a range of Impact Printing apparatus and for conciseness, we will present the two main milestones (Fig. 2):

*Milestone 1: front-end-loaded soft particles gun* (Fig. 2B)*:* the first iteration of the material shooting apparatus was a simple aluminum tube with a compressed air inlet connected to its back end. The soft particle loading was automatically performed by a “pick and place” procedure where the robot iteratively picked material by stamping a sheet of clay (Fig. 2B-12)—and moved to the propelling positions to shoot it. A range of prototypes were aggregated with this apparatus, however, the robotic arm kinematic motion to pick and shoot material proved to be inefficient both in terms of construction speed and material waste. Additionally, critical deviations were observed during the projectile flight, leading to the constraint of a 30-mm shooting distance, to ensure repeatability to the process. Such deviations can be explained by the absence of channeling during the acceleration phase: by loading the clay particle directly at the barrel's front end (Fig. 2B-3), this first prototype failed to provide the needed channeled acceleration for the projectile to gain a coaxial trajectory.

*Milestone 2: extruder-mounted soft particles gun* (Fig. 2C)*:* Consecutive steps were made toward a more efficient, precise, and waste-free Impact Printing apparatus. The latest prototype consisted of a clay extruder directly mounted onto a barrel and eliminated the robotic kinematic motion to pick material. Digital control enabled the extrusion of material directly into the shooting tube and activation of a piston compacted the cylindrical projectiles into the acceleration chamber (Fig. 2C-7). The projectiles were loaded at midbarrel to enable a channeled acceleration and a higher impacting precision (Fig. 3).

### Software setup

*CAD model and robot communication:* The digital blueprint is generated by Python and Grasshopper 3D scripts in a Rhinoceros 5.0^14^ CAD environment.

*Model discretization:* Contrary to conventional extrusion-based AM methods, Impact Printing does not require a continuous printing path for production. The computationally designed parts are divided into discrete shooting frames allowing for control over the sequence of the fabrication process.

The presented prototypes are built following a conventional running brick bond pattern. The digital model is translated into a set of shooting targets, defined as digital frames, characterized by a position and orientation in space. A linear offset along the *Z* axis defines the robot shooting position and distance.

*Robot communication:* All robot movements and actions are commanded by a custom-made Rhinoceros 5.0/Grasshopper 3D to UR5 communication platform. The robotic control manages the overall process, it interprets the digital blueprint and translates it into robotic positions.^15^ Such software interface allows both the design and robotic control to be embedded in a singular digital environment. It enables flexibility and adaptability in the digital design and fabrication processes.

### Aggregates' size and geometry definition

The cylindrical soft particles used for the experiments measure 10 mm diameter × 15 mm height and weight ∼2.2 g. Their shape and diameter are informed by the barrel, whereas their height (*D*_h_) is a multiple of their diameter (*D*_d_) such as *D*_H_/*D*_d_ ratio equals to 1.5. This maximized value results from previous experiments conducted to assess the optimal *D*_H_/*D*_d_ ratio: higher ratios were discarded resulting in clogging in the shooting apparatus and buckling behaviors upon impact.

### Experimental methodology

To characterize the fundamental Impact Printing process parameters and the effect of variable shooting pressures over the aggregations, we produced a range of standardized prototypes where all digital design and fabrication parameters are constant, except the variable propelling air pressure: 1.0, 3.5, and 5.0 bars.

The prototype dimensions fit in a 150 mm *W* × 100 mm *D* × 150 mm *H* bounding box and count *n*. 3 *W* × *n*. 5 *D* × *n*. 5 *H* soft particles. We use a layer-by-layer building sequence and three distinct material colors to mark the distinction between the individual soft particles in the aggregated mass. Finally, we selected the front-end-loaded soft particle gun to produce the parts as it allows for the simultaneous handling of various clay colors and the shooting distance was restricted to 30 mm to avoid deviations.

## Results

### Formal characteristics

The produced prototypes showcase distinct formal characteristics that contrast with conventional robotic processes. Typically, a machine's repetitive execution of a fabrication code ensures the production of precise and identical parts. Here, however, all parts differ as the material malleability coupled with shooting tolerances result in unpredictable deformations; the soft particles are compacted to different ratios and we observe some randomized horizontal shifts. Thus, the predictability of the robotic process contrasts with the unpredictability of the impacted soft particles' formal characteristics. As with research conducted on the aggregation of concrete drops,^16^ Impact Printing allows computational design and planning to control the overall form, but not the morphological details of the aggregated soft particles, resulting in the production of unique parts (Fig. 1).

### Impacting/impacted soft particles

We observe that upon impact, both impacting and impacted soft particles plastically deform. The impacting bodies seem to play a significant role in the building process as they compact and ram the already aggregated, but still malleable, mass. The impacting energy accumulates over the construction leading to the lower layers being consistently denser than the top layers. Various parameters such as impacting force, drying rate, construction sequencing and timing influence the result, for example, a soft particle impacting a hard surface will morph to the impacted form (Table 1). This ability leads to greater building freedom compared with conventional AM technologies often necessitating a controlled and flat printing support. Impact Printing can accommodate rough, uncalibrated or doubly curved base surfaces with no additional sensing required. The combination of the material morphing ability and the remote deposition process naturally compensates for tolerances.

**Table 1.** Comparative Table of the Soft Particles' 2D Morphologies—Extracted from the Prototypes Center Plane (Fig. 4)—Shot from a Constant Distance (30 mm) with Variable Air Pressures: 1.0, 3.5, and 5.0 bars

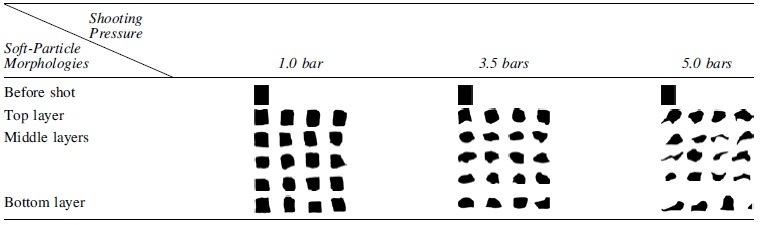

Soft particles 2D outline before propulsion, in top layer, in middle layers, and in bottom layer.

### Shooting force and bonding strength

**FIG. 4.**
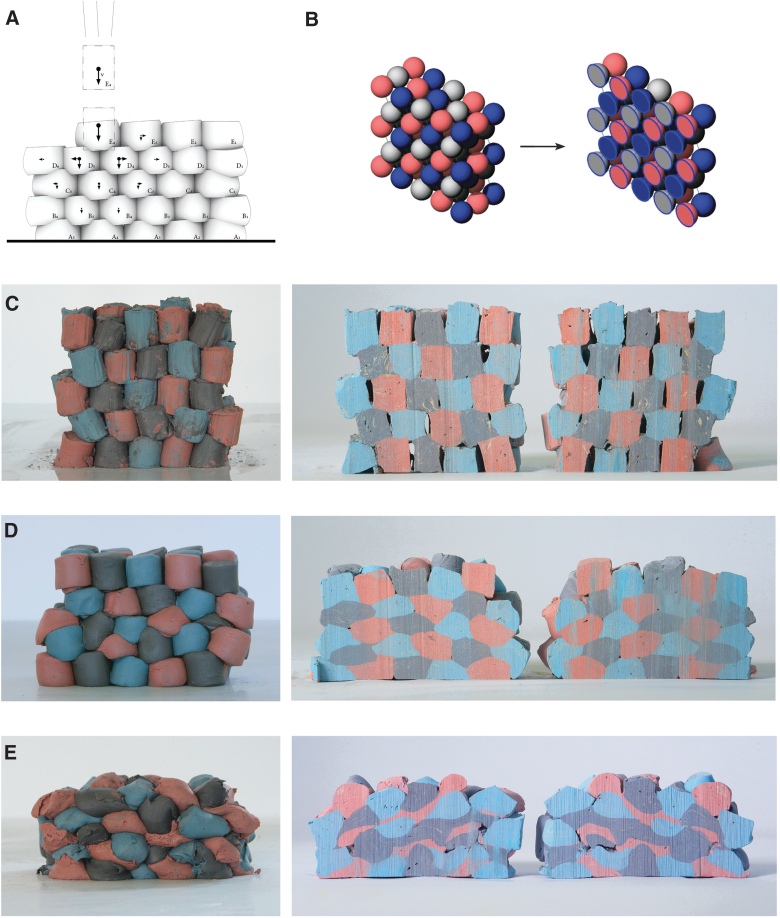
**(A)** Schematic of prototypes' impacting sequence, **(B)** Schematic of cut prototypes' color scheme, Impact Printed prototype built with: **(C)** low pressure—1.0 bar, **(D)** medium pressure—3.5 bars, **(E)** high pressure—5.0 bars. Image © Gramazio Kohler Research, ETH Zurich. Reprinted with permission.

Impact Printing has a unique formal vocabulary expressing both the soft particles' malleability and their impact. We observe a direct relation between the impacting force and the built formal outcome (Fig. 4): lower impacting forces result in porous aggregations with uncompacted soft particles, whereas high impacting forces result in compact masses. Such graded characteristics have both esthetics and structural consequences visible by analyzing the aggregations' bond.

To analyze the bond's formal details, we cut the prototypes in half to visualize their inner architecture (Fig. 4B) and observe the following: *Lower propelling pressure (1.0 bar)* results in no interlocking bond between the soft particles (Fig. 4C). They are stacked onto one another, with no morphological deformation, nor compaction (Table 1). As with a dry stacked brick bond, the structure is porous and the bond relies on the soft particles' surface cohesion not to fall apart. In this prototype, the amount of *E*_K_ transferred upon impact is too low to induce sufficient stress in the soft particle to plastically deform upon impact. The resulting prototype has low structural strength.

*Medium propelling pressure (3.5 bars)* results in a denser structure where the building blocks are compacted (Fig. 4D) to ∼50% of their initial height. The shot soft particles morphologically deform upon impact (Table 1), and a stronger bond emerges. A few air gaps remain in the built mass.

*High propelling pressure (5.0 bars)* results in a highly compressed aggregation (Fig. 4E) and the soft particles are compacted to ∼30% of their initial height. The soft particles, initially cylindrical, are morphologically deformed into aleatoric shapes and a complex interlocking bond emerges (Table 1). The high compaction ratio leaves nearly no air gaps in the aggregation and the overall prototype's height is the lowest of all samples.

### Preliminary mechanical testing

To evaluate the Impact Printing bond's strength, we proceed to the mechanical testing of prototypes. Typically, in layer-based 3D printing techniques, the structural weakness of the produced parts emerges at the interface between layers.^17^ Impact Printing could potentially overcome such constraint and use the impacting force to form a strong bond between the soft particles. In this experiment, our hypothesis would be confirmed by fractures lines running within—rather than in-between—the aggregated soft particles.

**FIG. 5.**
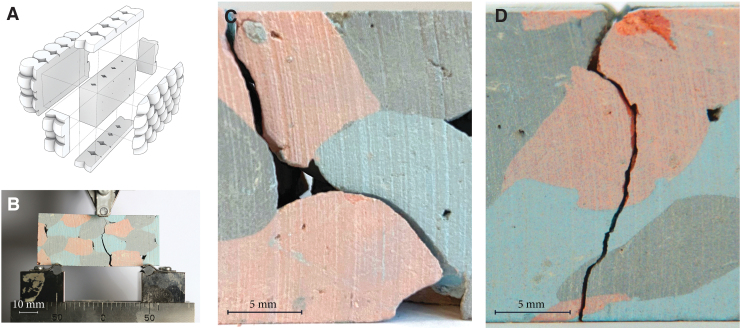
**(A)** Schematic characterization of tested parts. **(B)** Three-point bending test on prototype. **(C)** Detail of fracture path. medium bound prototype. **(D)** Detail of fracture path. highly bound prototype. Image © Gramazio Kohler Research, ETH Zurich. Reprinted with permission.

We cut the dry prototypes in rectangular 3-point testing bars measuring ∼10 mm *D* × 20 mm *H* × 60 mm *L*. Testing span = 50 mm (Fig. 5A). They comprise *n*. 5 elements in X, *n*. 2 elements in Y and *n*. 3 elements in Z, to read the fracture lines' propagation. The flexural behavior is tested using a Shimadzu AGS-X equipped with a 3-point bending fixture.

Only the prototypes showcasing medium and high compaction ratios were tested; the samples with the lowest compaction did not withstand the cutting process, proving a lower bonding between the soft particles. The testing of a part compacted to ∼50% resulted in a fracture line running mostly at the interface between the aggregates (Fig. 5C). The air gaps and low bonding between the aggregates lead to a rapid failure of the test specimen. In this sample, the kinetic energy transferred upon impact was too low to create a homogeneous and strong connection. In contrast, the fracture line of the sample with higher compaction seems to run independently to the soft particles interface. On the shown face (Fig. 5D), the crack starts in the middle of a soft particle and propagates vertically into the tested mass. This result seems to validate that, if aggregated with sufficient force, Impact Printing can potentially overcome the conventional AM interlayer weakness.

### Optimized Impact Printing setup

The improvement made to the Impact Printing apparatus led to an increased precision. The first iteration of the front-end-loaded soft particles' gun limited the shooting distance to 30 mm to minimize deviations. Effectively, for projectiles measuring 15 mm height, the actual flying distance was thus restricted to 15 mm—or 100% of soft particle height (sfh). This distance results from a series of tests to measure the deviations: a shooting distance of 20 mm (34% sfh) resulted in negligible deviations, whereas longer distances increasingly lead to shots fully missing their target,^18^ thus considerably limiting the potential of this AM process. The extruder-mounted soft particles' gun enabled the shooting distance to be doubled to 70 mm (367% sfh), thus extending the building work range of the impact printing apparatus (Fig. 3) and led to an improved cycle time by eliminating the kinematic motion needed to pick material.

## Discussion

The prototypes and observations presented in this article set a base for future experiments. They prove that structures can be produced by combining a robotic linear shooting process with soft particles. We observed that the material softness, propelling air pressure, and resulting impacting force are entangled and must be calibrated with one another to ensure a successful production. Thus, the data presented in this article are only valid for the selected material softness, robotic setup, and scale. Softer and firmer material would, respectively, require lower and higher amounts of energy to be aggregated. Therefore, Impact Printing interlaces materiality and robotic process.

We showed that Impact Printing can aggregate sustainable paste-like materials, such as clay. The material selection plays a significant role as the printing process uses its cohesion and morphing capacities as sole binding agent. For future experiments, the correlation between material and the energy required to permanently alter its form should be measured to extract clearer guidelines.

We proved that Impact Printing has the flexibility to build parts with both low compaction ratios, leading to porous structures, and high compaction ratios, leading to compact and homogeneous structural masses. Therefore, Impact Printing has the potential to produce graded structures by simply modulating the acceleration force.

Finally, we presented an Impact Printing apparatus that can build prototypes in the physical work range of the robot. The steps taken to improve the building range and precision were successful and extended the building work range of the robot. Additionally, the automatic loading of the material optimized the cycle time and limited the material waste. We believe both the Impact Printing speed and work range can be improved in future experiments. Spring or mechanical alternatives to the propelling mechanism should be explored to optimize reloading time and energy consumption, as well as to avoid some observed limitations, such as the clogging of the barrel.

## Conclusion

Impact Printing as a novel AM method is valid across a range of scales. The result obtained by aggregating clay—a scalable and widely used construction material—validates that Impact Printing of earthen compounds has potential for an architectural implementation. For architectural application a significant scaling-up of the impact printing apparatus is necessary and, consequently, fundamental digital fabrication parameters, including the soft particles' size, shooting force, and distance should be redefined altogether. On the material side, a novel earthen mix, suited to architectural purposes, should also be developed.

We envision a high-speed AM process calling for a reassessment of the traditional building element scale. Whereas standard architectural elements (e.g., bricks) were designed for the human hand and human work speed, a fast robotic fabrication process could enable a reduction of building block size, potentially resulting in higher resolution architecture exhibiting enhanced geometrical freedom.

Impact printing sequences fabrication into discrete steps and challenges the inflexibility of the conventional slicing-to-layering continuous AM process. The linear propulsion of material enables the material deposition process to be gravity free. A six-axis robotic setup allows Impact Printing to build in any directions or onto existing structures and thus open up new opportunities in computational design and digital fabrication. The direct correlation between the shooting force and the material compaction enables functionally and structurally graded architectural element (i.e., walls or columns), ranging from load-bearing masses to porous aggregations. Such material gradation can potentially seamlessly integrate ventilation or shading features. The material malleability coupled with the flexibility of the remote deposition process, could ease the integration of constructive systems such as reinforcements, services, and other building constraints that are typically challenging to integrate in an AM process.

Finally, in combination with sustainable material, we believe that a scaled-up and efficient Impact Printing method would make earth-based building processes economically more viable enabling new building scopes.

## Notes

^*^
The material mix provided by a local brick factory was composed of clay, sand, silt, lime, cellulose, wood chip and water.

^†^
For product extended composition, see product data sheet available at Bodmer Ton AG.
